# Generation and Research of Online English Course Learning Evaluation Model Based on Genetic Algorithm Improved Neural Set Network

**DOI:** 10.1155/2022/7281892

**Published:** 2022-10-11

**Authors:** Qiuping Song

**Affiliations:** Department of Foreign Language, North China University of Water Resources and Electric Power, Henan, Zhengzhou 450046, China

## Abstract

The rationality and timeliness of the comprehensive results of English course learning quality are increasingly important in the process of modern education. There are some problems in the scientific evaluation of English course learning quality and teachers' own English course learning, such as the need for proper adjustment and improvement. Based on the improved network theory of genetic algorithm, this paper takes an online English course learning quality evaluation model and uses MATLAB 7.0 to write the graphical user interface of the neural set network English course learning quality prediction model. The model uses the genetic algorithm of adaptive mutation to optimize the initial weights and values of the neural set network and solves the problems of prediction accuracy and convergence speed of English course learning quality evaluation results. Simulation experiments show that the neural set network has a strong dependence on the initial weights and thresholds. Using the improved genetic algorithm to optimize the initial weights and thresholds of the neural set network reduced the time for the neural set network to find the weights and thresholds that meet the training termination conditions, the prediction accuracy was increased to 0.897, the prediction accuracy was 78.85%, and the level prediction accuracy was 84.62%, which effectively promoted the development of online English course learning in colleges and the continuous improvement of teachers' English course learning level.

## 1. Introduction

The improvement of English course learning quality is an indispensable part of the modernization of national education, and the scientific, rational, and timely improvement of English course learning quality evaluation plays a key role [1–3]. However, the limitations of traditional English course learning quality evaluation methods have made them controversial. Therefore, it is necessary to establish a scientific and reasonable English course learning quality evaluation model to evaluate the teaching quality of undergraduate teachers in colleges and universities. At present, the teaching quality and English course learning quality evaluation system in colleges and universities need to be improved simultaneously, and an English course learning quality evaluation system suitable for the actual situation of colleges and universities needs to be constructed. Based on the above understanding, this paper will make the evaluation of English course learning quality more scientific and quantitative through intelligent technology. The prior guide samples are obtained by the entropy method, then the neural set network model is optimized to learn the prior sample knowledge by using the adaptive mutation genetic algorithm, and an evaluation model is established, which reduces the subjectivity of the neural set network learning samples [4–6].

With the rapid development of education informatization, it has been widely used in many aspects of higher education and achieved good results, but the research and practice of English course learning quality evaluation system and evaluation model to measure the quality of English course learning are relatively lagging behind. As we all know, the traditional English course learning quality model is not fully capable of solving such vague problems. The existing evaluation research at home and abroad mainly focuses on the following three aspects: evaluation subject, evaluation content, and evaluation method. According to the principle of constructing a perfect English course learning quality evaluation system, the paper first analyzes the advantages and disadvantages of the previous English course learning quality evaluation methods and then summarizes the problems existing in the current English course learning quality evaluation system in a certain university. The limitations of the teaching quality evaluation system should be improved to form a more scientific and reasonable English course learning quality evaluation system [7–9]. Based on the traditional genetic algorithm, a white adaptive mutation algorithm is proposed. The improved process is to use the adaptive mutation probability in the mutation operation process of finding the global optimal solution and use the adaptive mutation probability when the group obtains different fitness values and different mutation probabilities in order to achieve the purpose of enhancing or reducing the population diversity and the number of excellent individuals.

In this paper, the improved neural set network of genetic algorithm and self-organizing feature mapping neural set network are used for comprehensive evaluation and classification evaluation of English course learning evaluation system. It reflects the nonlinear characteristics of the system and makes the network have a strong generalization ability. Many of these different forms of indices involve the matching of the evaluation factor weights, and the value of the weights directly affects the evaluation results. The genetic algorithm, fuzzy technology, and neural set network are organically combined to form a genetic fuzzy neural set network. The general neural set network evaluation model membership function mainly adopts the normal type, but in the actual situation, the type and distribution of the membership function of the actual model cannot be determined. This paper uses the genetic algorithm to train the neural set network and then obtains the type and distribution of the membership function that conforms to the specific model of this paper, which can ensure the consistency of the type and distribution of the membership function with the specific model of this paper to the greatest extent. The fuzzy technology is then applied to the neural set network for optimization so that the selection of the neural set network has physical meaning.

## 2. Related Work

The neural set network has the ability of generalization and self-learning and can express complex nonlinear mapping within any range of accuracy while being able to learn knowledge from samples and abstract relevant laws [10–13]. In recent years, many researchers have adopted the neural set network method in the evaluation of English course learning quality evaluation model. Neural set networks are applied to the field of forecasting. Some scholars use the neural set network optimized by genetic algorithm to study the evaluation of quality, English course learning quality, and other fields, but few of them apply it to the field of English course learning quality evaluation [14]. This paper optimizes the evaluation model of English course learning quality in a university by combining genetic algorithm optimization neural set network, fully overcomes the above defects, and provides a more effective method for English course learning quality evaluation [15].

Żelasko et al. [16] enhanced the global search ability by introducing the “mutation” operation of the genetic algorithm and introduced a small probability random mutation to the position or velocity of the particle to enhance the diversity of the population (Dissipative PSO, referred to as DPSO) so that the algorithm can perform global searches more efficiently. Xin et al. [17] proposed to control the self-organized critical point by increasing the current critical value attribute of each particle. If the distance between two particles is less than a predetermined threshold, then increase the critical value of each other and redistribute their positions in the search space location to achieve population diversity. Cao et al. [18] proposed an improved particle swarm algorithm according to the problem that the basic particle swarm algorithm is easy to fall into the local optimum. Similar to the genetic algorithm, the SO algorithm adds a variation factor to help the particles escape from the local optimal solution. Experiments on five bellc breaking functions show that the VIIISO with variation factor can not only expand the search range of particles but also effectively speed up the convergence of the algorithm. Liu [19] proposed a new fuzzy particle swarm optimization algorithm FUZZY PSO. In this method, based on fuzzy set theory and the relationship between individuals and groups, the fuzzy control diversity method to overcome the lack of control population diversity was introduced, respectively.

As a general random search algorithm, SA has global asymptotic convergence, and its solution has nothing to do with the initial value. It is a parallel algorithm that converges to the global optimal solution with probability. In many practical applications, the simulated annealing algorithm is generally used to solve problems with high complexity, large scale, and little understanding of the field. Among the many optimization problems solved, Huang [20] has also achieved good results in English course learning problems. It is a good result, but people's research on English course learning problems has reached a certain field. Although the SA algorithm can solve some problems in English course learning, it cannot be combined with the existing algorithm knowledge. Management performance is the foundation and an important part of project performance. It mainly takes project managers as the subject of evaluation and dynamically checks, supervises, and evaluates their project management work [21]. The project and the manager must coexist at the same time, so the evaluation time point of management performance should cover the complete life cycle of the project from before to after, that is, the whole process evaluation. According to the specific content of performance evaluation, it is also composed of feasibility study report evaluation, evaluation report evaluation, decision approval evaluation, compliance evaluation (bidding, contract, capital use, cost, quality, etc.), and other parts. Heuristic random search algorithm is a more effective method to solve such complex optimization problems, and genetic algorithm is a heuristic search method. An important feature of this kind of algorithm is high robustness and wide application range. In the problem of English course learning, this group search algorithm can break through the limitation of field search and realize distributed information collection in the whole space, which may easily lead to the phenomenon of “prematurity” [22–25]. In practical applications, genetic algorithms are often used in combination with other algorithms, and the introduction of neural set networks can be considered to solve the problem that genetic algorithms are prone to premature convergence.

## 3. Improved Neural Set Network Hierarchy Based on Genetic Algorithm

### 3.1. Genetic Algorithm Adaptation

According to the established genetic algorithm adaptive grading index system and the collected data, first determine the specific structure of the neural set network (including the number of hidden layers and the number of nodes in each hidden layer), and then optimize the neural set network by establishing a genetic algorithm. Then transfer to the neural set network to train and learn the selected representative sample units to approximate the complex relationship between the English course learning impact factor and the natural quality score so as to complete the calculation of the natural quality score of English course learning.

It is a comprehensive simulation and embodiment of the complex system of the natural quality score of English course learning. Once the model of the network identifies the relationship between the English course learning impact factor and the natural quality score of English course learning through learning and gives the data of other graded units, the network can calculate the natural quality of other graded units through the fixed nonlinear mapping relationship point:

(1) $$\begin{array}{c} P\left[\frac{1}{t},\frac{2}{t},\frac{3}{t},\cdots ,\frac{n}{t}\right]=f\left(x|i=1,2,3\cdots ,n\right)\text{.} \end{array}$$

The basic principle of the traditional TOPSIS method is to use the vector normalization method to normalize the data after quantizing each index and establish a decision matrix. The TOPSIS method uses the Euclidean distance measurement scheme and sorts according to this distance. By analyzing the relationship between the positive and negative ideal solutions and the sorting rules, a reasonable scheme should be similar to the ideal solution, contrary to the negative ideal solution, and so on. Dimensionality reduction of learning samples is one of the main methods to improve the generalization performance of the network. Using principal component analysis to construct learning samples is an effective way to prevent and alleviate “overfitting” and improve the generalization performance of the network.

(2) $$\begin{array}{c} \log\left(pc\right)-\sum \exp\;\left(p\left(i\right)\right)\times \exp\;\left(p\left(j\right)\right)\times \exp\;\left(p\left(k\right)\right)=0\text{.} \end{array}$$

In addition, there are no special requirements for sample data. However, this method still has shortcomings; that is, when the index values of the two evaluation objects are in the middle of the optimal solution and the worst solution, accurate results cannot be obtained. The results show that this method can not only make up for the statistical method's low prediction accuracy and the complex and difficult realization of the numerical model method but also solve the problem that the training error of the unimproved neural set network is small, a new input, and a corresponding target. The output has a large error problem.

### 3.2. Neural Set Network Structure Division

For the determination of the weight of the neural set network structure, various methods have been proposed. Subjective weighting methods often rely on expert scoring and qualitative analysis, resulting in less accurate and more subjective results. The objective weighting method generally adopts the methods and techniques of mathematical statistics, which can objectively reflect the classification ability of mutual indicators and then determine the weights. There is a state function, namely, entropy; the increase of the entropy of the independent system must rely on irreversible processes, and the reversible process will not change the entropy of the independent system.

(3) $$\begin{array}{c} \begin{array}{c} H\left(t,t-1,t+1\right)\times {\left[H\left(t,t-1,t+1\right)\right]}^{-1}=\\ t\times \underset{n}{\sum }\left[\begin{array}{cccc} 1-f\left(x\right) & 2-f\left(x\right) & \cdots & y-f\left(x\right) \end{array}\right] \end{array}\text{.} \end{array}$$

The RBF is improved, and an adaptive basis function network (ARBF) model is established. The model can determine the number of hidden layer nodes according to the size of the network error until the error meets the requirements so as to avoid the hidden layer nodes in the network training process. The rationality of the comprehensive evaluation results of English curriculum learning quality can be accepted by school leaders, staff members of English curriculum learning management departments, and teachers. It plays an irreplaceable role in the development of colleges and universities, the cultivation of students, and the continuous improvement of teachers' English course learning level. The original materials can be used more fully, so the evaluation results are more in line with the actual situation, and the pros and cons of each evaluation object can be evaluated.

(4) $$\begin{array}{c} \frac{g\left(a\left(i\right),b\left(i\right)\right)+g\left(a\left(i\right),b\left(i\right)\right){}^{2}+g\left(a\left(i\right),b\left(i\right)\right){}^{3}}{\sum \exp\;\left(a\left(i\right)\right)*\exp\left(b\left(i\right)\right)}-\frac{1}{\log\;\left(g\left(i\right)\right)}=0. \end{array}$$

In the PCI model, the influence of the flatness of English course learning is not reflected, and it is only a function of the surface damage of English course learning. The advantage of this method is that it can accurately calculate and convert the overall damage caused by multiple damage. Moreover, the single-item repeated damage correction coefficient is used in the formula, which can avoid the phenomenon that the total deduction is too high or too low when evaluating a certain type of English course learning segment with more prominent damage. In theory, it should be relatively complete and rigorous, but the difficulty of the expert system deduction method is the accurate estimation of the deduction value and the correction value.

**Figure 1:**
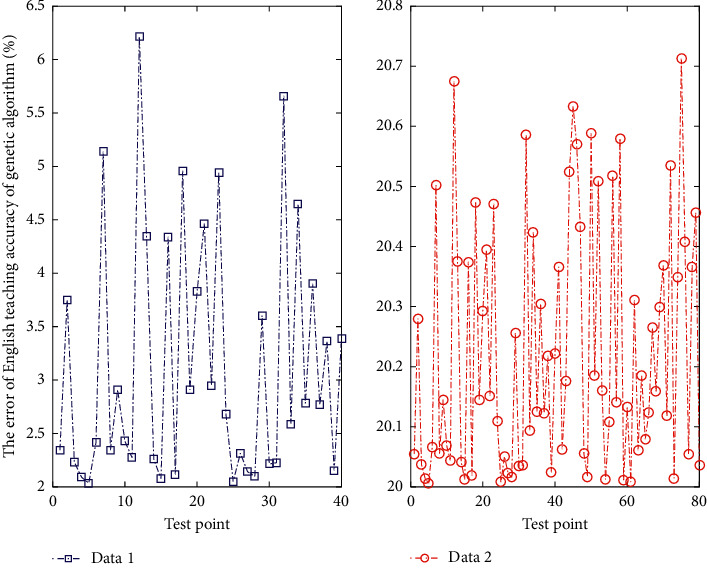
Comparison of English course learning accuracy of genetic algorithm.

Unsupervised learning is self-organizing learning, and the process of the network in Figure 1 is a self-learning process, which does not need to provide learning samples or external feedback. In the learning process, the network only needs to respond to the stimulus of the input signal, repeatedly adjust the network, and finally reach a certain orderly state to complete the network learning and training. The neural set network training based on supervised learning requires a certain number of training samples. Under this condition, the particle swarm will shrink statistically to the current global best position, more like a local algorithm.

### 3.3. Iterative Design of Neural Set Networks

**Figure 2:**
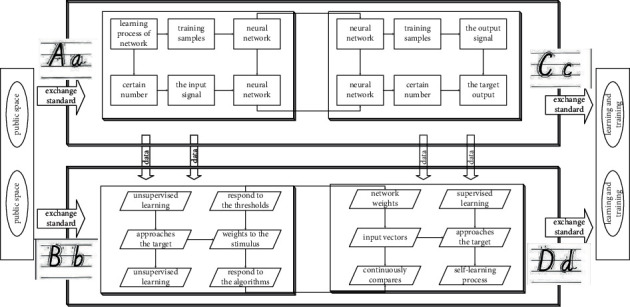
Iterative design process of online English course learning.

In this way, the neural set network continuously propagates in the two processes in Figure 2 until the results satisfy the requirements and output. In principle, English course learning problems can be solved by dynamic programming. It is just that since dynamic programming is a recursive solution, it means that a lot of space is needed to store intermediate results. Particularly before the problem of English course learning with a huge amount of data, there will be difficulties when using dynamic programming algorithm to solve the problem.

The grey relational analysis model is mainly used to study the uncertainty relationship between objects. It is a dimensionless operation that processes the data of the evaluation index and the degree of influence of factors on the target outcome. This method obtains the geometric figures through the data sequence and obtains the grey correlation degree through the similarity of the geometric figures. Generally speaking, the more similar the geometric figures are, the closer the correlation between the various factors is. The grey relational analysis method is a relatively simple and reliable analysis method. There is no requirement for the sample size, nor does it need to have a typical distribution law, and the amount of calculation is small.

(5) $$\begin{array}{c} \left\{\begin{array}{c} w\left(s,s\left(t\right)\right)\cap w\left(s,s\left(x\right)\right)=R\left(a,b\right),\\ w\left(x,s\right)=h\left(x,s\right),\\ h\left(x,x\left(s\right)\right)\cup h\left(s,s\left(x\right)\right)=\emptyset . \end{array}\right. \end{array}$$

Therefore, dynamic programming is generally only considered to be used when the amount of English course learning data is small. Each input to a processing unit has a relative weight, similar to the different synaptic strengths of biological neurons, with some inputs playing a more important role than others in generating a spiked output. In the network, the weights are adaptively adjustable coefficients that determine the strength of the input signal. Therefore, we also often regard them as a measure of connection strength. The initial weight of the processing unit can be improved and modified according to the rules of the network itself.

### 3.4. Algorithm Data Collection

When evaluating English course learning performance, the selection of algorithm data evaluation indicators should follow the following principles: (1) Purpose. The selected indicators should have a clear purpose and reflect the relevant content. The selected indicators should cover the evaluation content as much as possible, screen indicators that can fully reflect the English course learning situation, and try to avoid the simple application of incomparable indicators or absolute indicators. (2) Operability. Evaluation methods and indicators should be combined with the specific situation of English course learning management in domestic English course learning so that the evaluation indicators are measurable or practical. (3) Objectivity. In the determination of the evaluation index value, the influence of human factors on the essence of the evaluation object should be minimized so that the obtained results or data are objective and verifiable.

(6) $$\begin{array}{c} \overset{n}{\underset{i,j}{\sum }}\begin{array}{c} x\times f\left(i,j\right)\\ x\times f\left(i-1,j-1\right) \end{array}-\overset{n}{\underset{i,j}{\sum }}\begin{array}{c} e\times f\left(i,j\right)\\ e\times f\left(i-1,j-1\right) \end{array}=\left\{\begin{array}{c} x+e\left(i,j\right)\\ x-e\left(i,j\right) \end{array}\right.\text{.} \end{array}$$

The selection of network parameters such as the number of hidden layers, the number of hidden layer units, and the learning rate is generally determined by empirical formulas or continuous experiments, and the optimal parameters in Table 1 are finally selected according to the experimental results. Therefore, when faced with more complex problems, the network learning time will be longer, and the efficiency will be lower.

The neural set network structure planning is generally used to solve obvious problems in basic English course learning such as sorting, the shortest English course learning path, equipment updating, resource allocation, inventory management, and loading. English course learning problems are complex sorting problems. The advantage of dynamic programming is to decompose complex problems.

According to the established index system, from the perspective of AHP, structural performance evaluation can be divided into three basic levels: component, structure, and structural system. There are two fuzzy evaluation models for English course learning structure performance evaluation: single-factor fuzzy evaluation model and multifactor fuzzy evaluation model. The processing unit must evaluate the input signals to determine the strength of each signal, which is represented by the connection weight; secondly, it must calculate the “sum value” of all input signals, compare it with a certain limit level, and finally determine the processing unit. The output number of the processing unit is also only one like the biological neuron. The output unit gives the effect of the neural set network system on the external environment, and the number of nodes in the output layer is also determined according to the actual problem. Generally speaking, the ability of the neural set network to solve problems is very strong. But for a given structure of the network, its ability to solve problems is limited, and the more complex (or larger) the network, the better the problem-solving ability.

## 4. Construction of Online English Course Learning Quality Evaluation Model Based on Genetic Algorithm and Improved Neural Set Network

### 4.1. Improved Neural Set Network Fitting Branch

The neural set network evaluation index system is divided into four levels: the overall target *u* (one target), the evaluation criterion layer 0 (3 criteria), the damage category layer *D* (4 categories), and the evaluation index layer *E*(2 indicators). In this analytic hierarchy process model, the relationship between the factors at the upper level and the factors at the lower level is to formulate a questionnaire for arranging and judging elements. Through expert consultation, each judgment matrix is constructed, and the computer is used to perform hierarchical sorting and total hierarchical sorting calculation to obtain each evaluation. The cooperation between the modules of the system draws on the management model of assembly line operations.

(7) $$\begin{array}{c} \log\;\left\{\alpha k\left(ai,t\right)+\alpha k\left(ai-1,t-1\right)+\alpha k\left(ai-2,t-2\right)+\cdots +\alpha k\left(0,0\right)\right\}=\\ \left\{\begin{array}{c} \log\;f\left\{i,j\right\},i<j\\ \log\;2,i>j. \end{array}\right. \end{array}$$

The model includes the flowchart of the neural set network and the adaptive mutation genetic algorithm, and the optimal solution searched by the genetic algorithm is input into the neural set network as the initial weight of the network. Among them, the data flow of the model is as follows. The data information processing part starts from the genetic algorithm part of the adaptive mutation and determines the solution that meets the stopping condition through the steps of coding, fitness calculation, and genetic operation, that is, the optimal weight and threshold. After the BP neural set network obtains the initial weights and thresholds, it can reduce the time it takes to find the optimal weights and thresholds, thereby speeding up the convergence speed of the network. When the learning error of the sample or the number of iterations meets the requirements, a better AGA is obtained. The training and testing parts of the model have fully proved that the adaptive mutation genetic algorithm can improve the neural set network convergence speed and error accuracy.

(8) $$\begin{array}{c} \langle \begin{array}{c} \cap_{i=1,j=1}^{i+j}CORR\left(t-2,t-2\right)+CORR\left(n,m\right)=0,\\ \cap_{i=1,j=1}^{i+j}CORR\left(t-1,t-1\right)-CORR\left(m,n\right)=1. \end{array} \end{array}$$

The procedures to generate a function, calculate the difference, judge the convergence condition, and correct the function value are as follows:

- *Step 1*. Obtain a new solution belonging to the solution space by generating a function.
- *Step 2*. Calculate the difference between the new solution and the objective function. Since the function difference is only generated by partial transformation, it can be calculated by incremental calculation.
- *Step 3*. According to the acceptance criterion, judge whether the new solution meets the requirements. In quasi-side selection, the Metropolis criterion is most commonly used.
- *Step 4*. When the new solution is judged to be “adopted,” correct the value of the objective function with the transformed part of the current solution corresponding to the time when the new solution is generated.

At this time, one iteration has been realized, and the next iteration can be realized on this basis. If the new solution is determined to be “abandoned,” no operation is performed, and the current solution directly enters the next iteration.

**Figure 3:**
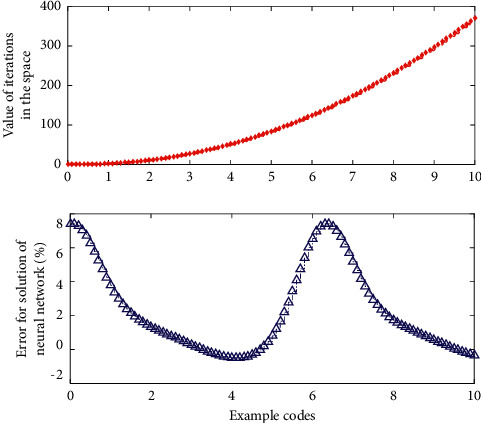
Iterative accuracy analysis of the new solution of the neural set network solution space.

Figure 3 shows that, in view of the ambiguity and ambiguity in the performance evaluation of English course learning, the fuzzy set theory is introduced into the English course learning evaluation, and the fuzzy evaluation set is established. The membership function of the set; through the membership function and the measured indicators of English course learning, the performance of English course learning can be evaluated, and finally, the comprehensive evaluation matrix of the performance evaluation of English course learning can be obtained.

### 4.2. Analysis of Quality Factors of Online English Course Learning

The rationality of English course learning methods and the quality of English course learning attitudes directly affect students' enthusiasm for learning. In the process of empirical research on the factors affecting effective English course learning in undergraduate classrooms in colleges and universities, of the different English course learning methods, the group discussion method is used by 80% of the college teachers who teach the course, 38.2% of the teachers can flexibly use the above different English course learning methods. There are 61.9% of teachers who can often choose appropriate English course learning methods, and teachers who use a single teaching method should learn to be flexible and adopt different teaching methods in order to stimulate students' interest in learning so as to improve students' learning efficiency.

(9) $$\begin{array}{c} \frac{\partial \left(e\left(t,i-1\right)\right)}{\partial \left(e\left(t\right)\right)}+\frac{\partial \left(a\left(t,i-1\right)\right)}{\partial \left(a\left(t\right)\right)}+\frac{\partial \left(w\left(t,i-1\right)\right)}{\partial \left(w\left(t\right)\right)}-\eta =0. \end{array}$$

**Figure 4:**
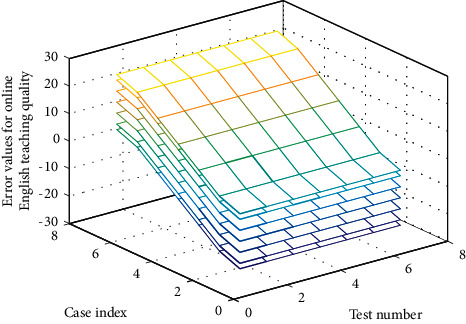
Three-dimensional analysis of online English course learning quality factors.

For a specific input mode, according to the basic principle of parallel inference of neural set network, each output node and its own signal are obtained by comparison, and other solutions can be excluded at the same time in this process. Then according to the comparison between the actual output and the target output, the traditional gradient descent optimization method in Figure 4 is used to reduce the error along the negative gradient direction of the error function.

The procedures to process training samples, design network topology, and determine network parameters are as follows:

- Organization of training samples selection and processing of training samples.
- Design of network topology, including the selection of the number of hidden layers, the number of hidden layer neuron nodes, and the transfer function of neuron nodes.
- Determination of main parameters of the network: determination of momentum coefficient, learning rate, and the number of learning times.

By selecting representative grading unit samples for learning, through the nonlinear mapping of the neural set network, the mapping relationship between the grading influence factor and the natural quality score is established, and this relationship is stored in the structure and weight of the network. In theory, the neural set network can learn the network with arbitrary precision; that is, it can achieve high-precision fitting of sample input and output. Once the neural set network model recognizes the relationship between the English course learning impact factor and the natural quality score of English course learning through learning and gives the data of other grading units, the network can calculate the natural quality scores of other grading units through association.

(10) $$\begin{array}{c} \frac{\partial T_{j}\left(i,j\right)}{\partial w_{j}\left(i,j\right)}=\frac{\partial a\left(i,j+1\right)}{\partial w_{j}\left(i,j\right)}-\frac{\partial b\left(i,j-1\right)}{\partial w_{j}\left(i,j\right)},if\left\{a-b>0\right\}. \end{array}$$

The quality of the training samples directly affects the connection weights and thresholds between neurons in the network, as well as the parameters in the membership layer, and directly affects the realization of network functions, so a typical model that can accurately reflect the actual situation is obtained. Training samples are very important. The extraction of training samples can be obtained from examples or from theoretical calculations. No matter which method is used, the samples should be typical and can fully and accurately reflect the state of the knowledge to be expressed.

MBA did not find a globally optimal solution to this problem, so even though it requires fewer FEs, it is still worse than BSAISA. Unfortunately, the robustness of BSAISA on this problem is relatively weak; however, this paper focuses on evaluating the performance of BSAISA in terms of convergence speed and local mining ability. The comparison results show that the FEs required by BSAISA to find the global optimal solution are less than those of other compared algorithms; that is, there is an obvious advantage in the convergence speed of the algorithm.

In the English course learning algorithm, although the traditional genetic algorithm has the global search ability, if there are individuals with excessive fitness, the probability of their chromosomes being selected and replicated increases so that there are too many offspring in Table 2, eventually leading to local convergence. Therefore, the genetic algorithm is often used in combination with the neural set network. In addition to increasing the comprehensibility of the model, the more important thing is that the neural set network can record the input variables in the database. In the early stage of training, the search range is relatively large, a larger learning rate can be selected, which does not fall into the local minimum value, and the training speed is fast; in the later stage of training, the search range is fixed near the global minimum value, which can be reduced by reducing the learning rate to ensure that the network converges to the optimal solution.

### 4.3. Analysis of Neural Set Network Topology

**Figure 5:**
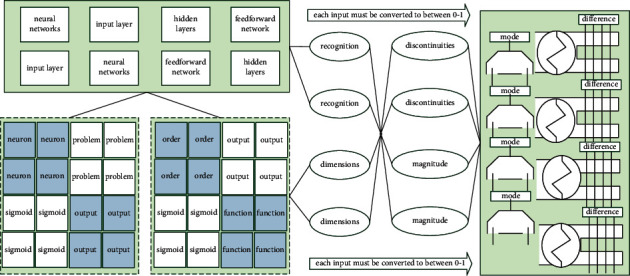
Neural set network hierarchy topology under genetic algorithm.

In the problem of English course learning, this group search algorithm can break through the limitation of field search and realize distributed information collection in the whole space. In the practical application of Figure 5, the genetic algorithm is often used in combination with other algorithms, and the introduction of a neural set network can be considered to solve the problem that the genetic algorithm is prone to premature convergence.

Neural set networks usually have one or more hidden layers. Generally, a single hidden layer feedforward network is called a three-layer feedforward network. The so-called three-layer includes an input layer, a hidden layer, and an output layer, and a single hidden layer network is used. The application is more common, and the hidden layer neurons usually use a sigmoid-type transfer function, while the output layer neurons use a purelin-type transfer function. Theory has proved that a three-layer neural set network, when the number of hidden layer neurons is large enough, can approximate any nonlinear function with finite discontinuities with arbitrary precision. In order to solve this problem, it is often necessary to compress the input samples of the network; when the multidimensional input samples belong to different dimensions and the order of magnitude difference is large, the values of each input must be converted to between 0 and 1; that is, normalization is performed to avoid affecting the recognition accuracy of the network due to the difference in magnitude.

(11) $$\begin{array}{c} \left\{\begin{array}{c} \lim\;i\left(t\right)=t\left(1\right)+t\left(2\right)+,\ldots ,+t\left(s\right),\\ \lim\;j\left(t\right)=\sqrt{t\left(1\right)+t\left(2\right)+,\ldots ,+t\left(s\right)},\\ \lim\;k\left(t\right)=\sqrt[{3}]{t\left(1\right)+t\left(2\right)+,\ldots ,+t\left(s\right)}. \end{array}\right. \end{array}$$

The set of time is *T* = {*T*1, *T*2, *T*3,…, *T* − *t*}, *T* − *i* is the time slice, and 0 < *i* ≤ *t*. The time slice refers to the time required by the teacher to complete a class; here, two classes are defined as one time. The three consecutive courses are represented as 1.5 time slices, so there are 42 time slices in a week. Of course, not all time slices are suitable for English course learning; as shown in the following figure, Saturday, Sunday, and evening 11-12 periods are unconventional English course learning times, and classes are not arranged as much as possible. The time slices used in regular English course learning are Monday to Friday during the day and 9-10 in the evening, a total of 25 time slices.

It can play a considerable role in the extraction of image texture features, and it has not yet been applied to the field of image retrieval; while the related feedback technology improves the retrieval accuracy, the degree of user participation is too high, and it cannot be automatically completed by the computer, which is not conducive to the image. In this paper, a texture feature extraction method based on M-band lifting wavelet is proposed and used for image retrieval, and a feedback verification image retrieval optimization algorithm based on secondary retrieval is proposed. The combination of the two algorithms becomes an effective texture image retrieval method.

In the model of the neural set network in Table 3, all functions and parameters of the network refer to the standard BP algorithm. The initial weight of the network in the index area A is [−1, 1], the training function is selected as train, the learning rate *r* is 0.01, the maximum number of iterations epochs is 10000. Since the index area B network is different from the index area A, its training parameters are slightly different from that of the index area A. At 12, the convergence speed is slow, so increase the maximum number of iteration epochs to 20000. According to the BP algorithm and the training function and training parameters of the neural set network, code is written in MATLAB (R2006b), and the training sample set is input for network training. The hardware environment in which the program runs is AMD Athlon (tm) 64 processor 3000Gmemory.

## 5. Application and Analysis of Online English Course Learning Quality Evaluation Model Based on Genetic Algorithm Improved Neural Set Network

### 5.1. Genetic Algorithm Data Preprocessing

Except for GA-DT and PSO, most algorithms are able to find the optimal function value of 0.012665 for this problem. In terms of FEs, BSAISA only needs 9440 FEs to reach the global optimum, which is better than most algorithms and second only to MBA's 7650 FEs. However, BSAISA's worst, mean, and Std values are better than those of MBA. In addition, they show the convergence of BSAISA and BSA on this problem, where *F*(*x*^∗^) on the *y*-axis is 0.012665. As shown in the figure, both BSAISA and BSA are trapped in local optima in the early iterations, but they are able to escape successfully again quickly. In addition, the convergence speed of BSAISA is significantly faster than that of BSA.

(12) $$\begin{array}{c} \frac{2}{3}f\left(w,t\right)*\left[x_{1}^{3}-x_{2}^{3}+y_{1}^{3}-y_{2}^{3}\right]=\frac{1}{3}\underset{x,k}{\sum }{\left(d\left(x_{1}\right)+d\left(k,x_{1}\right)\right)}^{3}\text{.} \end{array}$$

The structural method assumes that the texture of the image is composed of texture primitives arranged in a regular form. Then, extracting texture features is transformed into determining texture primitives and analyzing the arrangement rules of texture primitives. Statistical methods describe texture features through the random properties of grey-level distribution in images. This comparative experiment mainly compresses the image retrieval optimization algorithm based on feedback verification and the nonfeedback algorithm. Due to the difficulty in implementing the image retrieval algorithm based on user feedback and training feedback, the gap with this experiment is mainly reflected in the degree of user participation, so it was not carried out.

Through the comparison in the previous section, we found that the 3-band wavelet is better than the 2-band wavelet in terms of retrieval performance, so this section mainly discusses the comparison experiment between the feedback verification algorithm based on secondary retrieval and the nonfeedback algorithm under the 3-band wavelet decomposition. After the signal processing method transforms the original signal in the frequency domain, the frequency characteristics are counted in the transform domain to describe the texture features. With the development of the times, the four texture feature extraction methods penetrate each other and become an organically combined whole.

(13) $$\begin{array}{c} \left\{\begin{array}{c} \begin{array}{c} x_{1}^{3}-x_{2}^{3}=\left(y_{1}^{3}-y_{2}^{3}\right)\left(f|\left(x_{1}\right)|+|g|\left(k|,|x_{1}\right)\right){}^{3},\\ x_{1}^{3}-x_{3}^{3}=\left(y_{1}^{3}-y_{3}^{3}\right)\left(f|\left(x_{1}\right)|+|g|\left(k|,|x_{1}\right)\right){}^{3}, \end{array}\\ x_{2}^{3}-x_{3}^{3}=\left(y_{2}^{3}-y_{3}^{3}\right)\left(f|\left(x_{2}\right)|+|g|\left(k|,|x_{2}\right)\right){}^{3}. \end{array}\right. \end{array}$$

**Figure 6:**
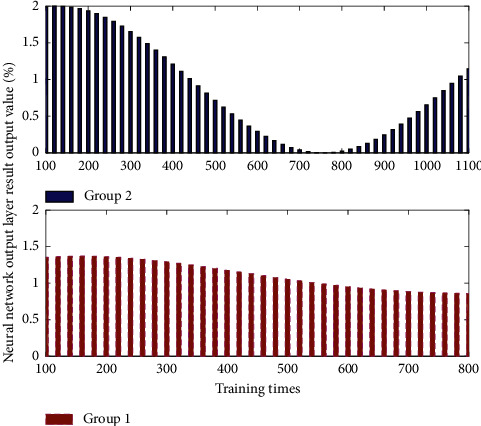
The distribution of the output value of the output layer of the neural set network.

Before optimizing the genetic algorithm, it is necessary to first determine several important parameter values of the genetic algorithm: crossover probability Pc, mutation probability Pm, and termination algebra *T*. These three parameters determine the result of the genetic algorithm calculation, but how to set these parameters has not been clearly explained so far in theory, and the empirical value or the empirical value under revision is generally used in practical operation. However, usually, a single neuron network cannot adapt to the complex English course learning needs of schools. In order to study the relationship between teachers, courses, and classrooms more thoroughly and get a more complete English course learning decision-making plan, we introduced a multilevel feedforward neural set network model. The network processing unit can be divided into input layer, hidden layer, and output layer. The input unit is used to accept external signals; the output layer realizes the result output; the hidden layer is located between the input layer and the output layer and is used to participate in the transition calculation. The neurons in different layers of Figure 6 are connected by connection weights, and the size of the connection weights reflects the connection strength between neurons.

This paper uses the trainscg training function provided by MATLAB, selects the sample data of 43 typical university engineering projects, and uses 500 groups of random sample data randomly generated by theoretical calculation to train the model. The target output is obtained by the fuzzy comprehensive evaluation method. The error target value is 101, and the maximum training times are 1000 times. After 343 iterations of the model, the error is 9.6324, which reaches the expected error target and meets the accuracy requirements, the convergence speed is fast, and the curve does not oscillate greatly.

Input parameters are course category, course nature, starting department, class time, class location, teacher's age, and teacher's gender. Output parameters are step coefficient: *α*, constraint coefficient: *β*, category evaluation coefficient: *γ*, and resource coefficient: *θ*. The English course learning class is obtained before the course arrangement and after the English course learning task arrangement is completed. The composition structure is as follows: (academic year semester) - course code - teacher's job number - serial number. The course code is an eight-digit string, the teacher's job number is a five-digit decimal code, and the serial number indicates the number of English classes a teacher teaches a certain course.

(14) $$\begin{array}{c} \left[\begin{array}{cc} f\left(w,d\right) & -f\left(b,d\right)\\ -f\left(-b,d\right) & f\left(w,b\right) \end{array}\right]=\left[\begin{array}{c} leader\left(x,d,b\right)\\ leader\left(x,w,b\right) \end{array}\right]*\left[\begin{array}{cc} fronter\left(\sin\;b\right) & fronter\left(\cos\;b\right) \end{array}\right]. \end{array}$$

In order to facilitate the calculation and storage of data, this two-dimensional table is converted into a one-dimensional vector for storage, and the converted one-dimensional description is shown in it. The column “1-1” represents the first lesson on Monday, and “4-3” represents the third lesson on Thursday. A value of “1” indicates that there is an English course learning task, and a value of “0” is the opposite. Step length refers to the number of credit hours between courses. In the neural set network toolbox, the function trainbpx-0 adopts two strategies of momentum method and learning rate adaptive adjustment, thereby improving the learning speed and increasing the reliability of the algorithm.

### 5.2. Simulation Evaluation of Online English Course Learning Model

The evaluation index system of undergraduate English course learning level in ordinary colleges and universities is divided into 7 first-level indicators, 19 second-level indicators, and 44 observation points. Among the 44 observation points, there are 12 quantitative indicators (i.e., basic school-running conditions indicators), of which the main six are the representation of English course learning resources per student. This paper mainly studies these six: the student-teacher ratio, the area of administrative space for English course learning per student, the number of computers for English course learning per 100 students, the number of seats in multimedia classrooms and speech classrooms for 100 students, the value of English course learning and scientific research equipment per student, and the number of books per student.

(15) $$\begin{array}{c} \langle \begin{array}{c} f\left(k,x_{2}\right)=y\left(w,da+1\right)*\left(T\left(x_{n}\right)-T\left(x_{2}\right)\right),\\ g\left(k,x_{2}\right)=y\left(w,db+1\right)*\left(T\left(x_{n}\right)-T\left(x_{2}\right)\right), \end{array}for\left(n=1,2,3\ldots ,w\right). \end{array}$$

**Figure 7:**
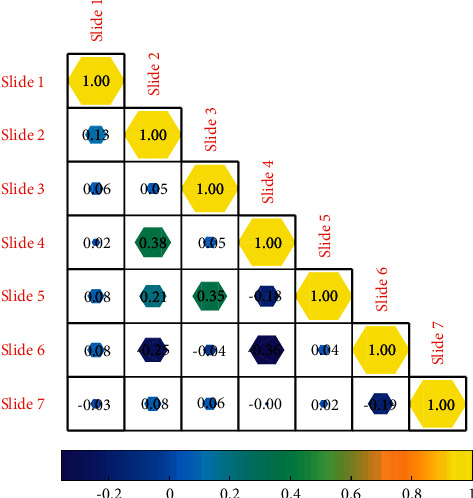
Systematic English course learning quality mapping under the constraint of neural set network.

It can be seen from the description of the algorithm that the particle swarm algorithm is a swarm intelligence algorithm that is very suitable for solving the above problem. On a feature subspace, the labeled datasets are first clustered according to the labeled classification (correlated and irrelevant). Generally speaking, the closer an unlabeled image feature vector is to the center of a similarly labeled cluster point, the higher the probability that it has the same label as the cluster. First, the primary random resource configuration parameter in Figure 7 is used as the dimension of each particle in the PSO algorithm, and the value range of each dimension is determined according to the *n* constraint relation in the actual situation.

Weak constraints refer to the conditions that need to be met as far as possible when teaching systematic English. There are many types of weak constraints, and it is often difficult to satisfy all of them (weak constraints often have conflicts in their own content). For example, teachers who are pregnant or have disabilities in legs and feet should give priority to the lower floors near the garage; teachers should try to arrange the floors closer to each other when they are connected to each other; after physical education classes, they are generally uneasy about English course learning activities in English course learning classrooms; public basic courses should be arranged in the morning, and so on. Weak constraints are determined by the teaching conditions themselves or put forward by teachers and students before English course learning and are obviously subjective and unpredictable.

(16) $$\begin{array}{c} \sum_{i=1}^{n}\ln\left(\sum_{j=1}^{n}\exp\left(g_{i},t\right)\right)+L\left(g\left(0\right),g\left(t\right)\right)-\sum_{i=1}^{n}y_{i}\left(f_{i}+\ln\sum_{j=1}^{n}\exp f_{i}\right)=0. \end{array}$$

Since pictures with the same label tend to have some consistency in image feature representation, the dataset can be expanded by taking advantage of this feature. The basic idea of this algorithm is as follows: firstly, the pictures in the database are grouped according to “positive correlation” and “irrelevance,” some unlabeled pictures that meet the conditions are labeled with pseudolabels, and finally, an estimation function is constructed to select a batch of appropriate pseudolabeled data for feedback model training. Then, the speed and position of each particle are randomly initialized according to the determined number of dimensions and the range of each dimension. When the number of iterations of the population reaches the specified value, or the fitness function reaches the lower limit, the algorithm ends, and the global extreme value *f* is the optimal solution, and its fitness function value is the optimal value.

### 5.3. Example Application and Analysis

The expert survey method is used for the evaluation of each indicator, and the score is based on 100 points or 10 points. The weighted evaluation value of the indicator is obtained by multiplying the indicator evaluation value and the normalized weight of the indicator. The inertia weight is used to control the exploration and development ability of the algorithm, and it is a balance factor to adjust the global search ability and local search ability of the algorithm. This feature fusion method is conducive to establishing the relationship between different image features in the encoding process and at the same time enhancing the expressiveness of the features. However, in the process of unified coding, this feature fusion method may have some additional requirements on the dimension and organization of different features; for example, the fused features are required to have the same dimension. Secondly, factors such as uneven distribution of image features may bring certain difficulties to the image encoding process.

In experiment 1 of this paper, the basic particle swarm algorithm 1 is used, and the evolution equation of the basic particle swarm algorithm is used to carry out the evolution. Firstly, the various underlying features of the image are represented by feature modeling, and the initial positions of the relevant feature points are retained during the feature modeling process. This image feature fusion method is adopted by the literature. In experiment 2, the particle swarm algorithm with inertia factor is used, and the method of linear decrease of inertia weight is used so that *σ* decreases linearly from 0.9 to 0.4 with the increase.

(17) $$\begin{array}{c} \left\{\begin{array}{c} \sum_{i=1}^{n}w\left(ai+1,bi\right)*\left(\sum_{j=1}^{n}\exp\left(g_{i},t\right)\right)=0\\ \sum_{k=1}^{n}v\left(aj+1,bj\right)*\left(\sum_{k=1}^{n}\exp\left(k_{i},x\right)\right)=0 \end{array}\right.,\left\{k,i,j=1,2,\ldots ,n\right\}. \end{array}$$

The independent variable is *s*, which is the decision variable *t*. The function *S*(*x*) is the number of students enrolled in the following year. *p* is the five English course learning resources, and *x* and *y* are the student-teacher ratio. There are six items in the value of scientific research equipment and books per student: *P* is the original basic resource value of the English course learning resource index of item *f* in year. *t* is the seventh year of a student in the *f*th item.

**Figure 8:**
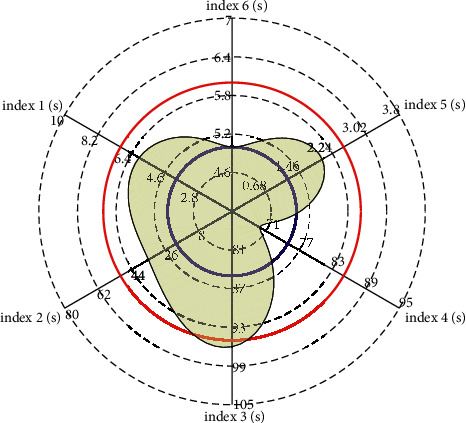
Radar chart of English course learning resource index evaluation.

How much standard resource value should be occupied. The mutation operation is an auxiliary search operation in the genetic algorithm, and its main purpose is to maintain the diversity of the population. In general, low-frequency mutation can prevent the possibility of losing an important single gene in the population, and high-frequency mutation will make the genetic algorithm into random search. The value of the general mutation probability is 0.1. Termination algebra *T* is a parameter that indicates the end of the genetic algorithm in Figure 8. The termination algebra in this experiment is 500.

In the actual English course learning, in addition to the English course learning “experience” (English course learning historical data), the factors that affect the English course learning decision-making are the established plans for this English course learning. In the English course learning algorithm studied in this paper, the neural set network will be used for the calculation of the fitness function of the genetic algorithm. The historical data of English course learning and the current English course learning plan that has converged are used as the input of the neural set network, and the output of the neural set network is used as the calculation parameter to act on the fitness function, so as to obtain the purpose of influencing the decision-making. The data analysis results of the neural set network will provide the relationship between teachers, students, courses, classrooms, and time in English course learning.

After forecasting the English course learning quality of each day, the actual concentration of pollutants and meteorological factors of the day are added to the original learning samples, the oldest historical data are removed, new learning samples are formed, and network training is performed to change the network weights, and so on. Image feature extraction is the process of regularly organizing the original high-dimensional and discrete images and obtaining the description of the underlying features of the image through feature modeling. Image quantization is higher-level modeling and representation of image features after obtaining the low-level feature description of the image. Due to the different emphases of different underlying feature descriptions, a single underlying feature often cannot construct a complete description of the entire image information. The weights and thresholds of the network are continuously updated; the forecast results of these three models are evaluated by the mean absolute percentage error, the mean square percentage error, the average forecast accuracy, the APl subexponential forecast accuracy, and the grade forecast accuracy.

(18) $$\begin{array}{c} \left\{\begin{array}{c} WSM\left[p,t\right]=\frac{2}{mn}\overset{p}{\underset{m=1}{\sum }}\overset{t}{\underset{n=1}{\sum }}\sqrt{\left(xi+yi\right)}-\sqrt{\left(1-xi\right)\left(1-yi\right)},\\ WSM\left[p,s\right]=\overset{p}{\underset{m=1}{\sum }}\overset{t}{\underset{n=1}{\sum }}\sqrt{\left(sx-sy\right)} \end{array}\right.\text{.} \end{array}$$

These conditions include internal factors of things and external factors required by things. The purpose of establishing the project evaluation logical framework is to establish the logical relationship between the target levels based on actual data so as to analyze the efficiency, effect, impact, and sustainability of the project. The obtained results are in good agreement with the actual test results, indicating that the model can well simulate the complex relationship between the four indicators and the English course learning quality index.

The relationship between the five indicators of length and area and the maintenance cost of English course learning, using the trained model to predict the required maintenance funds in each district, through the analysis of the forecast results, shows that the model thinks that English course learning and methods are feasible. The experimental results show that the use of the particle swarm algorithm can optimize the scale of college enrollment and English course learning evaluation model and obtain a suitable resource allocation plan so as to achieve the premise of meeting the excellent standard of English course learning quality evaluation under the condition of limited resources to predict the maximum feasible enrollment size for the following year.

## 6. Conclusion

The evaluation of university construction projects involves various environmental effects of the project, and it is difficult for a single or single-layer index to fully describe the effect of the project. Therefore, it is necessary to decompose the overall goal into subgoals or functions according to the goals and the effects of different scopes of influence produced by the construction projects of colleges and universities and then construct indicators. In order to improve the training effect, the number of learning samples involved in training should not be less than the number of connection weights of the network. However, the information on university construction projects that can be obtained from practice is limited, so this paper uses a theoretical calculation to generate several groups of random sample data. Combined with the current status of national university construction project performance evaluation, a performance evaluation grade suitable for national university construction projects has been formulated. In this paper, the genetic algorithm is improved, the concept of the algorithm is extended to the ecosystem, and the English course learning ecosystem model is established so as to decompose the complex English course learning problem into multigroup optimization problems to solve. Based on the above algorithm ideas, this paper designs and implements an intelligent class scheduling system based on BP neural set network and GA algorithm. The system selects ORACLE 10 as the database and uses JBuilder in the J2EE development environment to complete the running and debugging work. After the hierarchical structure model is established, the affiliation of each factor between the upper and lower layers is determined, and the problem is transformed into a sorting calculation problem in the hierarchy. In the ranking calculation, the ranking in each level can be simplified into a series of paired factors, judgment and comparison, and the judgment is quantified according to a certain ratio scale to form a comparison judgment matrix. The 1–9-scale method is generally used in the quantification of judgment, and the importance of each index is compared in pairs. Since the autoencoder has high computational complexity and no quantization function, it can not adapt to the problem of ultra-long sampling compression of audio signals. A self-encoder deep neural network based on convolutional wide sensing field is constructed to extract features of audio pulse modulation coding, and the drift problem in the data training process is prevented by combining with batch standardization. Then, combining genetic algorithm, fuzzy theory, and neural set network, the performance evaluation model of genetic fuzzy neural set network is constructed by using genetic fuzzy neural set network technology and MATLAB software. [26].

## Figures and Tables

**Table 1:** Optimal parameter distribution of neural set network.

Serial number	Network accuracy	Index system	Evaluation model	Problem-solving ability
1	1.24	1.3	1.36	1.42
2	0.71	0.73	0.75	0.77
3	0.18	0.16	0.14	0.12
4	0.35	0.41	0.47	0.53
5	0.88	0.98	1.08	1.18
6	1.41	1.55	1.69	1.83

**Table 2:** High-precision fitting of English course learning samples.

Training samples	Mean squared error (%)	Squared error (%)	Theoretical calculations	Quality of the training	Connection weights (%)
V 1	24.09346	53.15309	Redistribute their search location	Variation factor	16.09678
V 2	25.43208	54.77281	The distance between two particles	Comprehensibility of the model	16.03368
V 3	25.68576	55.07977	An improved particle	Swarm algorithm	16.55782
V 4	25.17601	54.46297	Increasing the current	Critical value	17.64163
V 5	24.27066	53.3675	A predetermined threshold	Local convergence	19.13484

**Table 3:** Iterative realization of genetic algorithm in neural set network.

Code number	Genetic algorithm in neural set network	Text content
1	Slices are suitable for *w*_*j*_(*i*, *j*)	#include <algorithm>
2	*ds* *du* *dθ* not all time goes	#include <functional>
3	During the day	Using namespace std;
4	The set of sin *θ*	Sort (a, a + 5, less <int>());
5	lim *i*(*t*) as shown in	Sort (a, a + 5, greater <int>());
6	A total of *P*(rea de r)	Template <class T> inline int
7	Each experiment was *f*(*w*, *t*)	Int a = {1, 4, 3, −13734, 1e3};
8	*x*_2_^3^ − *x*_3_^3^ of iterations epochs	Qsort (a, 5, Greater <int>);
9	The neural set network is *da*+1	Qsort (a, 5, Less <int>);
10	Which the *T*(*x*_*n*_) program runs	Fill (a, a + 105, 0);
11	The initial weight of *ai*+1	Fill (a, a + 105, 0x7fffffff)
12	*g*(*k*, *x*_1_) in the index area	#include <cstring>
13	The neural set network in table	Char s1 = “Hello,” “World;”
14	All *f*(*k*, *x*_2_) functions	Using namespace std;
15	In the model of g_*i*_	String s1, s2 = “World;”
16	The maximum number *x*_1_^3^	Freopen (“in.in,” “r,” stdin);
17	Unconventional English course learning	Freopen (“out.out,” “w,” stdout);
18	Parameters of the learning rate	Fclose (stdin); fclose (stdout);
19	Refer to *T*(*x*_*j*_) − *T*(*m*)	Fprintf (out, “%d,” a);

## Data Availability

The data used to support the findings of this study can be obtained from the author upon request.

## References

[1] Wu W. (2022). Neural Set Network Algorithm for English Course Learning Evaluation. *Innovative Computing*.

[2] Xing W., Zhang J., Zou Q., Liu J. (2021). Application of gauss mutation genetic algorithm to optimize neural set network in image painting art teaching. *Computational Intelligence and Neuroscience*.

[3] Mohamed M. A. (2022). A new federated genetic algorithm-based optimization technique for multi-criteria vehicle route planning using ArcGIS network analyst. *International Journal of Pervasive Computing and Communications*.

[4] Majumdar A., Jindal A., Arora S., Bajya M. (2022). Hybrid neuro-genetic machine learning models for the engineering of ring-spun cotton yarns. *Journal of Natural Fibers*.

[5] Bird J. J., Wanner E., Ekárt A., Faria D. R. (2020). Optimisation of phonetic aware speech recognition through multi-objective evolutionary algorithms. *Expert Systems with Applications*.

[6] Shen Y., Liu W., Wu Q., Chen R., Liu K. (2019). Leveraging neural network for online learning performance prediction and learning suggestion. *International Symposium on Emerging Technologies for Education*.

[7] Jiang Y., Zhang J., Chen C. (2018). Research on a new teaching quality evaluation method based on improved fuzzy neural network for college English. *International Journal of Continuing Engineering Education and Life Long Learning*.

[8] Zhang C., Guo Y. (2021). Retracted article: mountain rainfall estimation and online English course learning evaluation based on RBF neural set network. *Arabian Journal of Geosciences*.

[9] Duan J., Gao R. (2021). Research on college English course learning based on data mining technology. *EURASIP Journal on Wireless Communications and Networking*.

[10] Chen Y. (2021). Business English translation model based on BP neural set network optimized by genetic algorithm. *Computational Intelligence and Neuroscience*.

[11] Cai Y., Wang X., Xiong L. R. (2021). Difference analysis of regional economic development based on the SOM neural set network with the hybrid genetic algorithm. *Computational Intelligence and Neuroscience*.

[12] Saini J., Dutta M., Marques G. (2021). Fuzzy inference system tree with particle swarm optimization and genetic algorithm: a novel approach for PM10 forecasting. *Expert Systems with Applications*.

[13] Albadr M. A. A., Tiun S., Ayob M., Al F. T (2019). Spoken language identification based on optimised genetic algorithm–extreme learning machine approach. *International Journal of Speech Technology*.

[14] Deng X., Gu Y., Li F., Liu X., Zeng G (2021). Evaluation of teaching quality of computing method course based on improved BP neural network. *Journal of Physics: Conference Series*.

[15] Dogadina E. P., Smirnov M. V., Osipov A. V., Suvorov S. V (2021). Evaluation of the forms of education of high school students using a hybrid model based on various optimization methods and a neural network. *Informatics*.

[16] Żelasko D., Książek W., Pławiak P. (2021). Transmission quality classification with use of fusion of neural network and genetic algorithm in Pay&Require multi-agent managed network. *Sensors*.

[17] Xin X., Li-na W., Chun-ying Q. Teaching quality evaluation of “data structure” courses based on principal component analysis and support vector machine.

[18] Cao Y., Fan X., Guo Y., Li S., Huang H. (2020). Multi-objective optimization of injection-molded plastic parts using entropy weight, random forest, and genetic algorithm methods. *Journal of Polymer Engineering*.

[19] Liu B. (2021). Product appearance design and concept innovation based on neural set network. *Journal of Physics: Conference Series*.

[20] Huang Y. (2020). Research status and applications of nature-inspired algorithms for agri-food production. *International Journal of Agricultural and Biological Engineering*.

[21] Liu Z. T., Xie Q., Wu M., Cao W. H., Mei Y., Mao J. W (2018). Speech emotion recognition based on an improved brain emotion learning model. *Neurocomputing*.

[22] Qin R. Interactive effect evaluation model based on neural set network in the guiding of online ideological education.

[23] Viet D. T., Phuong V. V., Duong M. Q., Tran Q. T. (2020). Models for short-term wind power forecasting based on improved artificial neural network using particle swarm optimization and genetic algorithms. *Energies*.

[24] Song Y., Jiang T. Teaching quality evaluation method based on multilayer feedforward neural set network.

[25] Kiran M. S., Siramkaya E., Esme E., Senkaya M. N. (2022). Prediction of the number of students taking make-up examinations using artificial neural networks. *International Journal of Machine Learning and Cybernetics*.

[26] Mikolajczyk T., Moldovan L., Chalupczak A., Moldovan F. (2017). Computer aided learning process. *Procedia Engineering*.

